# Assessment of upper airways measurements in patients with mandibular skeletal Class II malocclusion

**DOI:** 10.1590/2177-6709.20.5.086-093.oar

**Published:** 2015

**Authors:** Nayanna Nadja e Silva, Rosa Helena Wanderley Lacerda, Alexandre Wellos Cunha Silva, Tania Braga Ramos

**Affiliations:** 1 Specialist in Orthodontics, Associação Brasileira de Odontologia (ABO-PB), João Pessoa, Paraíba, Brazil; 2 Coordinator, Postgraduate Program in Orthodontics, Associação Brasileira de Odontologia (ABO-PB), João Pessoa, Paraíba, Brazil; 3 Professor, Postgraduate Program in Orthodontics, Associação Brasileira de Odontologia (ABO-PB), João Pessoa, Paraíba, Brazil; 4 Professor of Orthognathic Surgery, Postgraduate Program in Orthodontics, Associação Brasileira de Odontologia (ABO-PB), João Pessoa, Paraíba, Brazil

**Keywords:** Angle Class II malocclusion, Oropharynx, Nasopharynx, Airway obstruction

## Abstract

**Objective:**

Mandibular Class II malocclusions seem to interfere in upper airways measurements. The aim of this study was to assess the upper airways measurements of patients with skeletal Class II malocclusion in order to investigate the association between these measurements and the position and length of the mandible as well as mandibular growth trend, comparing the Class II group with a Class I one.

**Methods:**

A total of 80 lateral cephalograms from 80 individuals aged between 10 and 17 years old were assessed. Forty radiographs of Class I malocclusion individuals were matched by age with forty radiographs of individuals with mandibular Class II malocclusion. McNamara Jr., Ricketts, Downs and Jarabak's measurements were used for cephalometric evaluation. Data were submitted to descriptive and inferential statistical analysis by means of SPSS 20.0 statistical package. Student's t-test, Pearson correlation and intraclass correlation coefficient were used. A 95% confidence interval and 5% significance level were adopted to interpret the results.

**Results:**

There were differences between groups. Oropharynx and nasopharynx sizes as well as mandibular position and length were found to be reduced in Class II individuals. There was a statistically significant positive correlation between the size of the oropharynx and Xi-Pm, Co-Gn and SNB measurements. In addition, the size of the nasopharynx was found to be correlated with Xi-Pm, Co-Gn, facial depth, SNB, facial axis and FMA.

**Conclusion:**

Individuals with mandibular Class II malocclusion were shown to have upper airways measurements diminished. There was a correlation between mandibular length and position and the size of oropharynx and nasopharynx.

## Abstract (pt)

**Introdução:**

as más oclusões de Classe II mandibulares parecem interferir nas dimensões das vias aéreas superiores. Assim, o objetivo do presente estudo foi avaliar as vias aéreas superiores de pacientes com Classe II esquelética, verificando a associação entre essas dimensões e a posição mandibular, o comprimento mandibular e a tendência de crescimento, comparando-os com um grupo pareado de pacientes com Classe I.

**Métodos:**

foram avaliadas 80 telerradiografias de perfil de 80 pacientes com 10 a 17 anos de idade, sendo 40 com má oclusão de Classe I e 40 com Classe II mandibular, pareados por idade. Para a avaliação cefalométrica, foram utilizadas medidas de McNamara Jr, Ricketts, Downs e Jarabak. Os dados foram submetidos à análise estatística descritiva e inferencial, por meio do*software* SPSS 20.0, utilizando-se os testes*t* de Student, coeficiente de correlação de Pearson e coeficiente de correlação intraclasse. Para interpretação dos resultados, adotou-se um intervalo de confiança de 95% e nível de significância de 5%.

**Resultados:**

houve diferença entre os grupos, e as medidas da orofaringe e nasofaringe foram menores no grupo de Classe II, assim como as medidas de comprimento e posição mandibular. Houve correlação positiva estatisticamente significativa entre a orofaringe e as medidas Xi-Pm, Co-Gn e SNB; já a nasofaringe apresentou correlação com as medidas Xi-Pm, Co-Gn, profundidade facial, SNB, eixo facial e FMA.

**Conclusão:**

indivíduos portadores de Classe II mandibular apresentaram as medidas das vias aéreas superiores diminuídas. Observou-se uma correlação entre o comprimento mandibular e a posição mandibular e as dimensões da orofaringe e da nasofaringe.

## INTRODUCTION

Skeletal Class II malocclusion is a dentofacial deformity caused by a growth disorder of the bones frequently associated with mandibular retrusion relative to upper facial structures.^1^ This deformity is also associated with functional disorders, mainly affecting upper airways and the temporomandibular joint.^2^^,^ 3

Patients with skeletal Class II malocclusion who have this deformity due to deficiency in mandibular growth present with a retrognathic mandible either because of growth vector or by deficient mandibular length.

According to Muto et al,^4^ craniofacial abnormalities, including mandibular retrognathism, short mandibular body length and backward/downward rotation, can lead to decreased pharyngeal airway. These findings indicate that nasopharyngeal obstruction may be related to changes in mandibular morphology.^5^

The study of upper airways and their relationship with mandibular position and size is extremely important in orthodontic diagnosis because of their association with obstructive respiratory disorders, especially sleep apnea. This knowledge is definitive to the indication of mandibular advancement, whether orthopedic or surgical, for treatment of these disorders.

Several studies have been carried out with a view to measuring the pharyngeal airway; however, comparison with Class I individuals and the correlation between the variables involved in Class II malocclusion and airways measurements are still scarce, which encouraged the present study.

## MATERIAL AND METHODS

This study was submitted to and approved by the Ethics Committee on Human Research through Plataforma Brasil, following the norms of the law 466/2012, under approval protocol #835.928. 

The sample comprised 80 digital lateral cephalograms belonging to 80 patients of both sexes, without associated abnormalities, aged between 10-17 years, with a mean age of 12.3 years, treated by postgraduate orthodontic students (ABO/PB, Brazil). Of the 80 images, 40 were from patients with mandibular Class II malocclusion, whose diagnosis was confirmed by Xi-Pm, Co-Gn, Go-Me, facial depth and SNB measurements (at least three of these measures should be reduced so that the image would not be withdrawn from the sample). The other 40 radiographs belonged to Class I individuals. Groups were matched by age.

**Figure 1:**
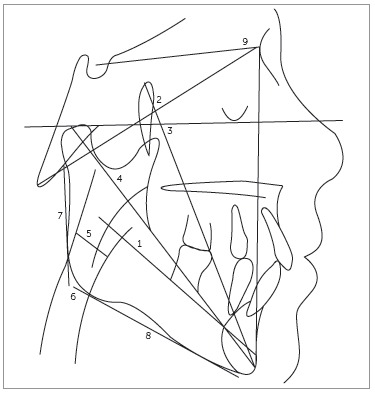
- Cephalogram and cephalometric measurements used.

Anatomical tracings of all radiographic images were made on acetate paper, in a dark room, by an examiner using graphite pencil (point 0.3). Each film was traced by one investigator and checked by a second one, so as to verify the accuracy of anatomical outline determination and landmark placement. Measurements of mandibular length and spatial position, as well as size of nasopharyngeal and oropharyngeal airways, were taken using the cephalograms (Fig 1, Table 1).

Measurements were taken twice, with a 10-day interval in between, with the aid of a millimeter ruler and a 180° protractor. The first assessment was carried out with the entire sample while the second one was carried out with 30% of the sample.

Procedures of statistical inference were performed based on parametric statistics. Correlation coefficient and intraclass correlation coefficient (ICC) were used to assess intraexaminer agreement. The choice for statistical test was based on normal distribution of data, according to Komogorov-Smirnov normality test (*p* > 0.05). Intergroup comparison was performed by Student's t-test and Pearson r correlation coefficient. For descriptive procedures, absolute and relative data and measurements of central tendency and variability were presented. A 95% confidence interval and 5% significance level (*p*< 0.05) were adopted to interpret the results. Data were submitted to SPSS 20.0 statistical package for Windows and analyzed by means of descriptive and inferential statistics.

**Table 1:** - Cephalometric measurements used.

**Measure**	**Clinical standard**	**Appropriate age**	**Description**
1. Xi-Pm	65 ± 3 mm	9 years (1.6/year)	Axis of the mandibular body - a line extending from point Xi to the mental protuberance.
2. Facial axis (Ba.NA x Frankfurt)	90 ± 3°	Does not change upon growth	Provides the direction of growth of the chin and the ratio between facial height and depth.
3. Facial depth (NA-Pog x Frankfurt)	87 ± 3°	9 years (0.33/ year)	Indicates the anteroposterior position of the mandible.
4. Co-Gn (Effective mandibular length)			Consists in the geometric relationship between the maxillomandibular length, directly linked either to patient's age or sex.
5. Oropharynx	10 to 12 mm		Measured by the width of the pharynx at the point where the posterior border of the tongue (in the radiograph) crosses the lower border of the mandible up to the posterior pharyngeal wall.
6. Nasopharynx	Mixed dentition: 12 mm Permanent dentition: 17.4 mm		It is measured linearly from a midpoint on the posterior wall of the soft palate to the posterior pharyngeal wall where there is the greatest closure of the airway.
7. Ar-Go	44 mm	11 years (male: 1.01 - 7.2) (female: 0.71 - 4.2)	Height of the mandibular ramus.
8. Go-Me	71 mm	11 years (male: 1.11 -7.11) (female: 0.73 - 3.12)	Length of the mandibular body.
9. SNB	80°		Anteroposterior position of the mandible in relation to the base of the cranium.

## RESULTS

In order to assess the reliability of measurements of oropharynx and nasopharynx, mandibular length, mandibular position and direction of mandibular growth, the examiner conducted two assessments which were followed by determination of intraexaminer agreement. This calculation was done using intraclass correlation coefficient (ICC). Results were statistically significant and indicated intraclass coefficients ranging from 0.97 (facial depth) and 1.00 (oropharynx), thereby denoting strong intraexaminer agreement (Table 2).

**Figure 2:**
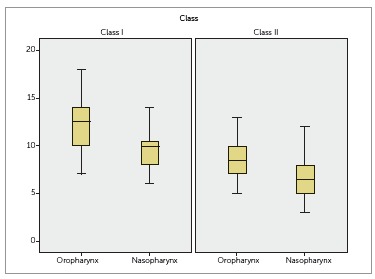
- Assessment of upper airway measurements of Class I and Class II groups.

As for upper airways measurements, statistically significant differences were found between both groups (*p* < 0.001). That is, the size of nasopharynx and oropharynx is reduced in Class II individuals (Fig 2).

**Figure 3:**
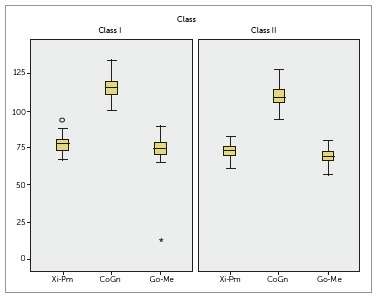
- Assessment of mandibular length of Class I and Class II groups.

The same results were observed for mandibular length, with significant differences between groups. The following measurements were found to be greater in Class I individuals: Xi-Pm, Co-Gn and Go-Me (Table 3, Fig 3).

**Figure 4:**
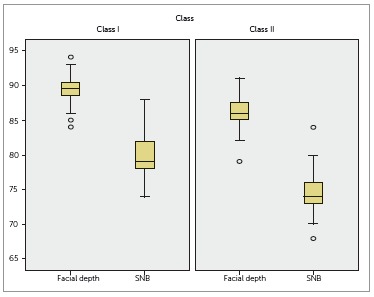
- Assessment of mandibular position of Class I and Class II groups.

As shown in Table 3, measurements of mandibular position also indicated significant differences between groups, with facial depth and SNB being greater among Class I individuals (Table 3). These results are graphically shown in Figure 4.

**Figure 5:**
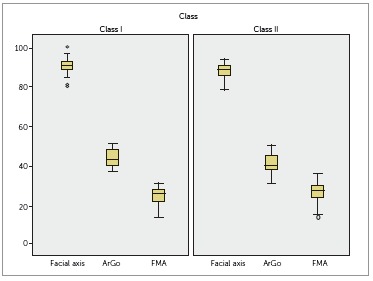
- Assessment of mandibular growth of Class I and Class II groups.

Measurements related to the direction of mandibular growth also differed significantly between groups. Facial axis and Ar-Go were greater in Class I individuals, while FMA was found to be greater in Class II individuals (Table 3, Fig 5).

In order to assess the correlation between oropharynx/nasopharynx size and mandibular length, position as well as growth, Pearson *r* correlation coefficient was performed.

Significant positive correlations were observed between the oropharynx and Xi-Pm, Co-Gn and SNB. Moreover, there were also correlations between the nasopharynx and Xi-Pm, Co-Gn, facial depth, SNB, facial axis and Ar-Go. Given that such correlations were positive, it is concluded that the greater the measurements of upper airways, the greater the variables, as reported herein. Correlation coefficients ranged from 0.24 to 0.37; thus, indicating weak to moderate correlations between variables (Table 4).

**Table 2:** - Assessment of intraexaminer agreement.

**Measures**	**ICC**	**p**	**Interpretation**
Xi-Pm	0.99	< 0.001	Strong intra-examiner agreement
Co-Gn	0.99	< 0.001	Strong intra-examiner agreement
Go-Me	0.99	< 0.001	Strong intra-examiner agreement
Facial depth	0.97	< 0.001	Strong intra-examiner agreement
SNB	0.99	< 0.001	Strong intra-examiner agreement
Facial axis	0.99	< 0.001	Strong intra-examiner agreement
Ar-Go	0.99	< 0.001	Strong intra-examiner agreement
FMA	0.99	< 0.001	Strong intra-examiner agreement
Oropharynx	1.00	-	Perfect agreement
Nasopharynx	0.99	< 0.001	Strong intra-examiner agreement

**Table 3:** - Assessment of upper airways measurements, mandibular length, mandibular position and direction of mandibular growth of each group.

**General measures**	**Specific measures**	**Class I**	**Class II**	**t (p)**		
		**Mean ± SD**	**Min-Max**	**Mean ± SD**	**Min-Max**	
Upper airways	Oropharynx	12.2±2.5	7 - 18	8.6±1.7	5 - 13	7.4 (< 0.001)
	Nasopharynx	9.4±1.9	6 - 14	6.7±1.9	3 - 12	6.2 (< 0.001)
Mandibular length	Xi-Pm	77.5±5.4	67 - 94	72.4±4.8	61 - 83	4.4 (< 0.001)
	Co-Gn	115.6±6.7	100 - 134	109.7±7.5	94 - 128	3.6 (< 0.001)
	Go-Me	73.5±11.2	13 - 90	69.4±4.9	57 - 80	2.0 (0.04)
Mandibular position	Facial depth	89.4±2.4	84 - 94	86.1±2.5	79 - 91	5.8 (< 0.001)
	SNB	79.7±2.9	74 - 88	74.5±2.9	68 - 84	7.8 (< 0.001)
Direction of the mandibular growth	Facial axis	90.5±3.7	80 - 100	87.4±3.6	78 - 93	3.8 (< 0.001)
	Ar-Go	43.9±4.1	37 - 51	40.5±4.8	31 - 50	3.3 (0.001)
	FMA	24.9±3.8	14 - 31	27.0±4.9	14 - 36	2.0 (0.04)

**Table 4:** - Correlation between upper airways measurements and mandibular length, position as well as direction of mandibular growth in both groups.

**Measures**	**r**	**P**	**%**	**Interpretation**
Oropharynx				
Xi-Pm	0.31	0.004	9.6%	Significant, positive and moderate correlation
Co-Gn	0.24	0.02	5.7%	Significant, positive and weak correlation
Go-Me	0.13	0.23	1.6%	There was no correlation between variables
Facial depth	0.21	0.06	4.4%	There was no correlation between variables
SNB	0.37	0.001	13.6%	Significant, positive and moderate correlation
Facial axis	0.12	0.26	1.4%	There was no correlation between variables
Ar-Go	0.12	0.28	1.4%	There was no correlation between variables
FMA	-0.07	0.52	0.4%	There was no correlation between variables
Nasopharynx				
Xi-Pm	0.37	0.001	13.6%	Significant, positive and moderate correlation
Co-Gn	0.32	0.003	10.2%	Significant, positive and moderate correlation
Go-Me	0.18	0.11	3.2%	There was no correlation between variables
Facial depth	0.29	0.009	8.4%	Significant, positive and weak correlation
SNB	0.34	0.002	11.5%	Significant, positive and moderate correlation
Facial axis	0.28	0.01	7.8%	Significant, positive and weak correlation
Ar-Go	0.29	0.007	8.4%	Significant, positive and weak correlation
FMA	-0.13	0.24	1.6%	There was no correlation between variables

## DISCUSSION

Although some recent studies have reported a need for tridimensional evaluation by magnetic resonance,^6^^,^ 7 ^,^ 8 its high cost and lack of standardization of patient's head position still hamper the use of this method for research. According to Muto et al,^9^ a change of 10^o^ in craniofacial tilt may affect measurement taking in the area of upper airways in approximately 4 mm. Lateral cephalograms have been used in this type of assessment as part of patients' basic orthodontic records, with the advantage of having low costs and low radiation dose, being of easy access, and providing standardization of measures with high reproducibility for diagnosis.^6^^,^ 10 ^,^ 11 These advantages render this method common in research,^7^^,^ 9 ^,^ 12 ^,^ 13 ^,^ 14 which validates the methodology adopted in the present study and allows comparison of results. The reproducibility of the method was confirmed statistically, with strong intraexaminer agreement.

The studied sample comprised patients aged between 10 and 17 years old, with a mean age of 12.3 years, similarly to other studies,^5^^,^ 12 ^,^ 15 ^,^ 16. Because there are minor changes in the nasopharynx as a result of growth,^17^ the sample was matched by age; thus, avoiding potential bias as regards data interpretation. In terms of sex, groups were similar, although we found three more males than females in the Class II group.

Regarding airways measurements, there were significant differences between groups, with Class I patients having oropharynx and nasopharynx greater in size (Table 3, Fig 2). These findings corroborate the majority of studies found in the literature.^14^^,^ 18 ^,^ 19 ^,^ 20 The studies by Freitas et al^12^ as well as Memon, Fida and Shaikh^21^ found no interference of malocclusion in oropharynx and nasopharynx width when they compared Class I to Class II patients. Differences in our results may be related to the methods employed, since those studies included a Class II sample based on dental occlusion and may have included subjects with Class II resulting from maxillary prognathism, whereas in our study, mandibular Class II was confirmed cephalometrically.

In order to have a better understanding of which factors inherent to malocclusion could be related to changes in upper airways, we initially diagnosed differences in skeletal features between groups, as follows: mandibular length (Xi-Pm, Co-Gn and Go-Me), mandibular position (facial depth and SNB), and direction of growth (facial axis, Ar-Go and FMA).

As regards mandibular length, measurements found in the Class I group were greater than those found in the Class II group (Table 3, Fig 3), thereby confirming mandibular Class II diagnosis. These data validate the assumption that mandibular length can be related to the size of upper airways, which is in agreement with Muto et al^4^ who pointed out that craniofacial abnormalities, including mandibular retrognathism, short mandibular body and downward rotation, may cause a decrease in the size of airways, as reported by other studies.^9^^,^ 13 ^,^ 19 ^,^ 22 ^,^ 23 The same behavior was observed in the variables related to spatial position of the mandible. As expected, the mandible in the Class II group was found retropositioned in relation to the cranial base when compared to the Class I group. This information allows us to conclude that both position and length of the mandible, i.e., the effective length of the mandible, must be considered in the diagnosis of patients with Class II malocclusion. Nevertheless, a greater or less interference of either one of these variables cannot be assumed. In the literature, this comparison is scarce and only cited by a few authors.^1^^,^ 23 ^,^ 24

Our study was carried out considering that several others have assessed the association between facial growth pattern and upper airways measurements.^5^^,^ 12 ^,^ 15 ^,^ 16 ^,^ 19 When comparing Class I and Class II groups, FMA and facial axis indicated an increased vertical trend among Class II individuals as well as a shorter mandibular ramus. According to Jarabak,^25^ this finding refers to mandibular morphology with a clockwise growth pattern. This same feature was reported in the study by Joseph et al^15^ who used a sample of individuals with Class II malocclusion. This information does not allow us to claim that all mandibular Class II individuals will have a vertical growth trend, although such feature was found in the sample. However, there seem to be an association between vertical pattern and reduced airways measurements, which has already been reported by several studies.^5^^,^ 12 ^,^ 14 ^,^ 19

The correlation between oropharynx and nasopharynx was studied separately from other variables, as shown in Table 4. There was a positive correlation between the size of the oropharynx and mandibular length, represented by Xi-Pm and Co-Gn, and the position of the mandible, represented by SNB. In agreement with our findings, studies carried out in the last five years^7^^,^ 20 ^,^ 23 ^,^ 24 have concluded that mandibular length and position influence airways measurements.

Although Class II malocclusion patients have mostly presented with a vertical growth pattern in relation to Class I individuals, our results could not support a correlation between vertical pattern and a shorter oropharynx. We did not observe a positive correlation between growth pattern measurements (FMA, Ar-Go and facial axis) and the size of the oropharynx, even though there was an association. This is in agreement with the reports by Castro and Vasconcelos.^16^ On the other hand, Freitas et al,^12^ Zhong et al^19^ as well as Ucar and Uysal^5^ found a correlation between growth pattern and the size of the oropharynx.

When assessing airways measurements and growth pattern, Joseph et al^15^ showed a correspondence between dolichocephalic individuals and shorter airways, particularly the nasopharynx. This is in agreement with our findings, as seen in Table 5 which shows a significant positive correlation between Ar-Go values and the size of the nasopharynx. In addition, they showed a positive correlation between Xi-Pm, Co-Gn, facial depth, SNB and facial axis; thus, concluding that mandibular length and position are related to the size of the nasopharynx.

Mandibular retrusion is one of the factors that may cause obstructive sleep apnea syndrome (OSA), characterized by a collapse site hindering the passage of air located in the pharynx. A reduction in this region can be the etiology of this syndrome both in children and adults. Characterized by respiratory disorders and nocturnal snoring, OSA may cause psychological and social impairment for the individual.^11^^,^ 22 ^,^ 23

As the results of our study suggest that mandibular length and position as well as the direction of growth can influence measurements of pharyngeal airways, we emphasize the importance of mandibular advancement in growing children through orthopedics by means of functional appliances; and in adults, with surgical advancement in order to promote enlargement of airways for functional and quality of life improvement, as well as decreased morbidity.^8^^,^ 13 ^,^ 14 ^,^ 26 ^,^ 27

## CONCLUSION

» Individuals with mandibular Class II malocclusion were shown to have upper airways measurements reduced when compared to Class I individuals.

» Mandibular length is related to a decrease in upper airways measurements. Similarly, anteroposterior positioning of the mandible exerts influence on airways measurements.

» There was a tendency of facial growth pattern with a positive, but weak correlation with the sizes of the nasopharynx, but not with the oropharynx.
